# Final thermal conditions override the effects of temperature history and dispersal in experimental communities

**DOI:** 10.1098/rspb.2014.1540

**Published:** 2014-10-22

**Authors:** Romana Limberger, Etienne Low-Décarie, Gregor F. Fussmann

**Affiliations:** Department of Biology, McGill University, 1205 Avenue Docteur-Penfield, Montreal, Quebec, Canada H3A 1B1

**Keywords:** global change, biodiversity, gradual, abrupt, metacommunity, species interactions

## Abstract

Predicting the effect of climate change on biodiversity is a multifactorial problem that is complicated by potentially interactive effects with habitat properties and altered species interactions. In a microcosm experiment with communities of microalgae, we analysed whether the effect of rising temperature on diversity depended on the initial or the final temperature of the habitat, on the rate of change, on dispersal and on landscape heterogeneity. We also tested whether the response of species to temperature measured in monoculture allowed prediction of the composition of communities under rising temperature. We found that the final temperature of the habitat was the primary driver of diversity in our experimental communities. Species richness declined faster at higher temperatures. The negative effect of warming was not alleviated by a slower rate of warming or by dispersal among habitats and did not depend on the initial temperature. The response of evenness, however, did depend on the rate of change and on the initial temperature. Community composition was not predictable from monoculture assays, but higher fitness inequality (as seen by larger variance in growth rate among species in monoculture at higher temperatures) explained the faster loss of biodiversity with rising temperature.

## 1. Introduction

Predictions on the effect of global change on future biodiversity are complicated by the multi-factor nature of global change, involving changes in CO_2_, temperature, nutrients, pH, precipitation patterns and the frequency of extreme events [1]. Indirect effects of global change via altered species interactions further complicate our capacity to predict the effects of global change [2,3]. In addition, the effect of climate change depends on habitat properties. Habitats differ in their current climatic conditions, in the rate and amount of change they are expected to experience, in the degree of connectivity with other habitats and in the heterogeneity of the landscape they are embedded in. While a number of experiments have tested for interactive effects of various global change stressors [4,5], the potential interaction of climate change with habitat characteristics has received far less attention.

Habitats have been predicted to vary in the rate and amount of warming, depending on latitude and on location relative to oceans and mountain ranges [1]. Although it has been shown for CO_2_ that an abrupt increase can have a stronger effect on diversity than a gradual increase [6], no such comparison has been made for the effect of different rates of rising temperature. A slower rate of environmental change increases the ability of organisms to respond by adapting to the changing conditions [7,8], which could mitigate a negative effect of environmental change on biodiversity. Furthermore, different environmental histories can set communities on different successional trajectories and have long-lasting effects on diversity and community composition [9].

Data on thermal sensitivities of species suggest that the effect of climate change on local biodiversity will depend on the current thermal conditions of the respective habitat [10,11]. In tropical habitats, ambient temperatures are close to or even above the thermal optima of species, while temperate and polar organisms experience average temperatures that are below their optimum temperature for growth [10,11]. Warming of already warm habitats should thus have a more detrimental effect on species than in cooler habitats, where warming could result in a fitness increase. However, the few studies that analysed interactive effects of warming and current climatic conditions within a community context found that current temperature had little or no effect on how warming affected diversity [12,13].

Given that many species respond to climate change by migrating to higher latitudes or altitudes [14], the effect of climate change on diversity will crucially depend on whether species are able to track environmental change by dispersal [15]. In addition to natural variation in habitat connectivity, changes in land use have resulted in an increase in habitat loss and fragmentation, which will probably exacerbate the negative effect of climate change [16]. In spatially heterogeneous landscapes, dispersal can increase local diversity by maintaining species in sink habitats owing to continuous re-immigration from source habitats [17] and by providing insurance against changing environmental conditions [18]. Experimental studies that analysed interactive effects of climate change and habitat fragmentation found some indication for dispersal to mitigate the negative effect of climate change on diversity [19,20] and to buffer ecosystem functions against the effect of warming [21]. However, there is also evidence for the effect of warming to be independent of dispersal [22].

Predictions on the effect of climate change on biodiversity usually ignore species interactions [23]. While some studies found that the response to temperature or CO_2_ within a community was at least partly predictable from single-species responses [24,25], a number of experiments suggest that competitive interactions alter species responses to climate change [2,26,27]. It remains to be determined whether a simple measurement such as performance of a species along a temperature gradient measured in isolation can be used to predict its response to rising temperature or whether aggregate measurements across separate species can contribute to our prediction of the effect of temperature on biodiversity.

In a microcosm experiment with phytoplankton communities, we manipulated factors that vary among regions and habitats: the rate of temperature change, initial and final temperature, dispersal and landscape heterogeneity. By manipulating a whole suite of habitat characteristics, we were able to determine their relative importance in structuring diversity. We tested the hypotheses that: (i) an abrupt increase in temperature would have a stronger effect on diversity than a gradual increase, (ii) warming would have a stronger effect in initially warm than in initially cool habitats by driving more species close to or above their limit of thermal tolerance, and (iii) warming would have a stronger effect in isolated than in connected habitats, in particular when landscapes are heterogeneous with respect to temperature. Alternatively, diversity would depend only on final temperature, irrespective of the rate of change and of the degree of dispersal, and the effect of warming would be of the same direction and magnitude both in cool and warm habitats. In addition, we analysed whether the response of species to temperature within the community was consistent with the response measured in a short-term monoculture experiment.

## 2. Material and methods

### (a) Model communities

We used artificially assembled communities comprising 10 algal species from four different taxonomic groups (electronic supplementary material, table S1). Culturing was done in growth chambers with light continuously provided at 100 µE m^−2^ s^−1^. Microcosms were 125 ml glass flasks filled with 50 ml of modified Bold's basal medium [24,28]. At the beginning of the experiment, we added all 10 species to each microcosm, with an initial biovolume of 500 000 µm³ ml^−1^ per species. Two steps of acclimatization and stabilization were performed. First, before the start of the experiment, monocultures of the 10 species were acclimatized for two weeks at the initial experimental temperatures of 20°C and 25°C, respectively. Second, the communities were maintained at the initial conditions (i.e. 20°C and 25°C, respectively) for 4 days before starting the dispersal and environmental change treatments.

### (b) Experimental design

We constructed landscapes consisting of two habitats (i.e. microcosms). We manipulated initial and final temperatures, environmental change, dispersal and landscape heterogeneity across the experimental landscapes (electronic supplementary material, figure S1). At the beginning of the experiment, each habitat was at one of two initial temperatures (cool: 20°C, warm: 25°C) and was then exposed to one of three temperature change treatments (constant, gradual, abrupt), resulting in three possible final temperatures (20°C, 25°C, 30°C). Temperature change was manipulated by exposing the flasks to a gradual or abrupt increase in temperature of 5°C. In gradually changing environments, temperature was increased by 0.2°C per day, starting on day 4 of the experiment and ending on day 28, while the abrupt increase was imposed on day 16. This difference in timing ensured that over the course of the experiment the mean temperature was the same for gradual and abrupt change. The communities were maintained for 56 days, such that each community was at its final temperature for at least 28 days before the experiment ended.

The two habitats of a landscape either had the same initial temperature (homogeneously cool or homogeneously warm landscapes) or differed in initial temperature (heterogeneous landscape). The two habitats of a landscape were either unconnected or connected by dispersal. To manipulate dispersal, we exchanged 1 ml of the culture volume between the two flasks of a connected landscape twice a week, giving a dispersal rate of 2% every 3.5 days. In isolated pairs of flasks, we also removed 1 ml of culture but placed it back into the same flask. All 18 treatment combinations were replicated four times, resulting in 144 microcosms.

### (c) Semi-continuous culturing and sampling

Cultures were maintained through semi-continuous culturing. Once a week, we removed 10% of the culture from each flask and replaced the withdrawn volume with fresh medium. On days 28 and 56, the removed volume was used for sampling. Samples were fixed with Lugol's solution and counted under an inverted microscope.

### (d) Monoculture growth experiment

We measured the intrinsic rate of increase of each species in monoculture at 20°C, 25°C and 30°C. Individual species were cultured in 48-well plates (Corning Inc.) after inoculating 1 ml of medium with 10 µl of the culture. Plates were sealed with sterile air-permeable membranes (Aeraseal by Excel Scientific Inc.) and continuously shaken at 400 r.p.m. After 5 days of acclimatization, 10 µl of each culture were used to inoculate fresh well plates, using three replicates per species. Over the course of 7 days, we measured absorbance at 660 nm twice a day on an optical plate reader (Synergy-HT, BioTek, Winooski, VT, USA) to estimate cell density.

### (e) Data analysis

We measured diversity by calculating species richness and evenness (*J*’ = *H*’/ln(*S*), where *H*’ is the Shannon index and *S* is species richness) at the scale of local patches and at the scale of the landscape and measured beta diversity by calculating the Bray–Curtis distance, BC, between the two patches of a landscape, with
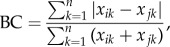
where *x* is the relative biovolume of species *k* in patches *i* and *j*. The BC distance is a dissimilarity index appropriate to measure beta diversity [29]. As it is a better measure of the ecological contribution of each species and is common practice in microcosm experiments with algae [30,31], we based the calculations of evenness and BC distance on the relative biovolume of the species.

We estimated the relative importance of the predictor variables in explaining diversity using random forests [32], a statistical modelling approach highly suitable for ecological data [33,34]. Predictors in our model are the type of change, initial temperature, final temperature, heterogeneity and dispersal. Random forests were computed with the RandomForest package in R [35]. See the electronic supplementary material for additional information.

We used generalized linear models (GLM) to explicitly test for significance of the predictors and for interactions among them. Effects on species richness, which is Poisson distributed, were analysed using GLMs with a log link function, effects on evenness were analysed with ANOVA, and effects on BC distances were analysed using GLMs assuming a quasi-binomial distribution and using a logit link function. Owing to the dependency of the observations at the scale of local patches, we computed the analyses for richness and evenness only at the scale of the landscape (mean of local diversity and regional diversity, respectively). As predictor variables we used change (constant, gradual, abrupt), dispersal (without, with) and landscape (homogeneously cool, homogeneously warm, heterogeneous). We calculated three-way GLMs (model: response ∼ change × dispersal × landscape) with separate analyses for the two sampling dates, with Bonferroni correction used to account for the repeated sampling. In addition, we analysed the effect of treatments on species composition using a MANOVA with the frequency of species calculated from biovolume as the response variables. Also, we analysed the effect of treatments on total biovolume.

We also analysed whether final temperature affected diversity and whether its effect depended on the temperature history using GLMs. Since our experiment was factorial with respect to initial temperature but not with respect to final temperature, we could test only for the main effects of final temperature and change when using the whole dataset. To test for an interaction between final temperature and temperature history, we conducted two separate analyses for final temperatures of 25°C and 30°C and tested whether temperature history affected diversity.

We estimated intrinsic rates of increase (*r*) and carrying capacities (*K*) of the species at 20°C, 25°C and 30°C by fitting logistic growth curves (*N_t_* = *KN*_0_/[*N*_0_ + (*K* − *N*_0_) exp(−*rt*)]) to the absorbance data from the short-term monoculture growth experiment. Because some of the slow-growing species had not reached full carrying capacity by the end of the experiment, we focus our discussion on growth rates. One of the species, *Cryptomonas* sp., did not grow in the well plates, and we thus do not have data on growth rates for this species. However, *Cryptomonas* was not found in any patch of the main experiment by day 28. All analyses were computed in R v. 2.15.3 [35].

## 3. Results

### (a) Relative importance of the predictor variables

**Figure 1.**
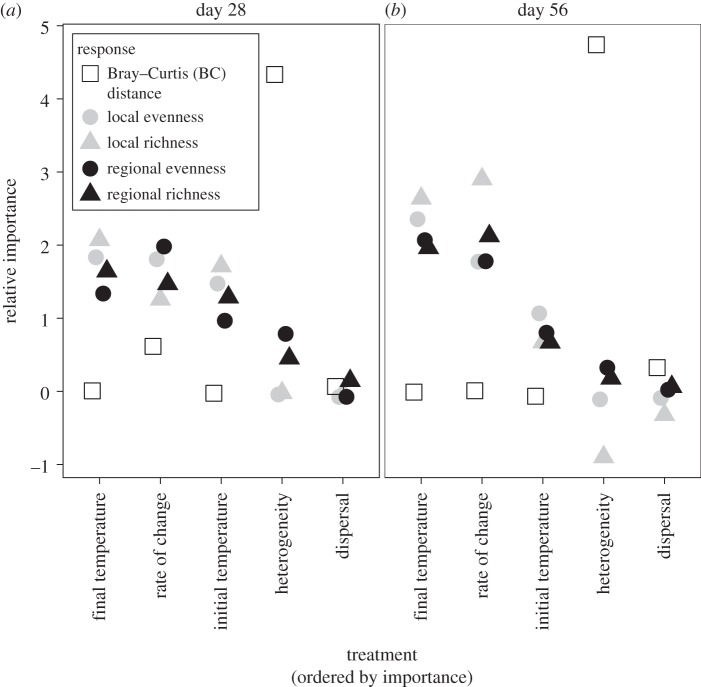
Importance of treatments for the biodiversity response at the patch and regional scale as determined using the random forest algorithm. Importance of the treatments was measured as the per cent increase in the mean squared error of the model for predicting the out-of-bag data when the treatment is omitted from the model. Final temperature and the rate of change were highly important in determining diversity. For species richness, the importance of rate of change is dominated by the contrast between constant and changing environments (figure 2). The exclusion of dispersal from the model does not affect its predictive ability. The BC distance is an exception to the importance of temperature; this measure of biodiversity is largely determined by landscape heterogeneity.

**Figure 2.**
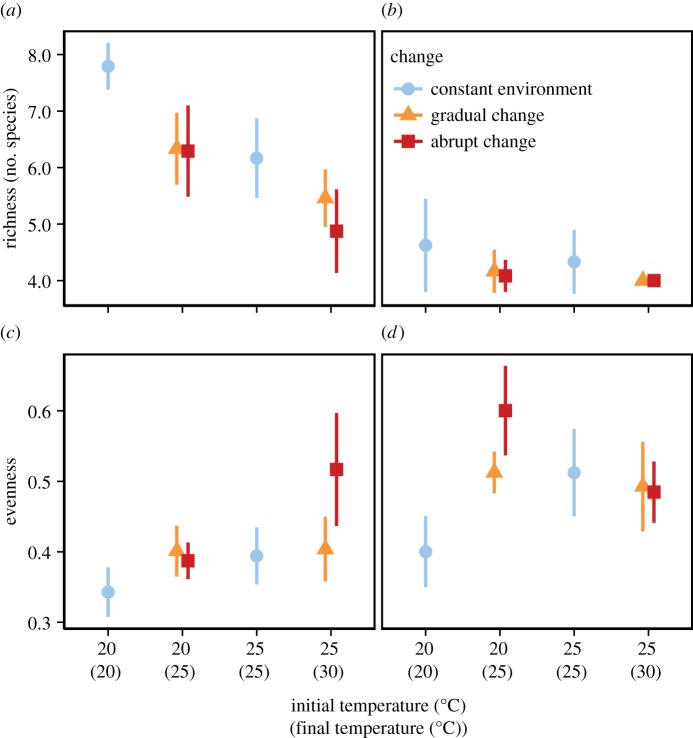
The effects of the rate of change, initial and final temperature on species richness (*a,b*) and evenness (*c,d*) on day 28 (*a,c*) and day 56 (*b,d*). Communities were either kept at a constant temperature or were exposed to a gradual or abrupt increase by 5°C. Initial temperature of the habitats was 20°C or 25°C, resulting in final temperatures of 20°C, 25°C and 30°C, respectively. Data from connected and unconnected habitats were pooled; points are the mean and bars are a standard deviation. *n* = 24 for each treatment combination. (Online version in colour.)

**Figure 3.**
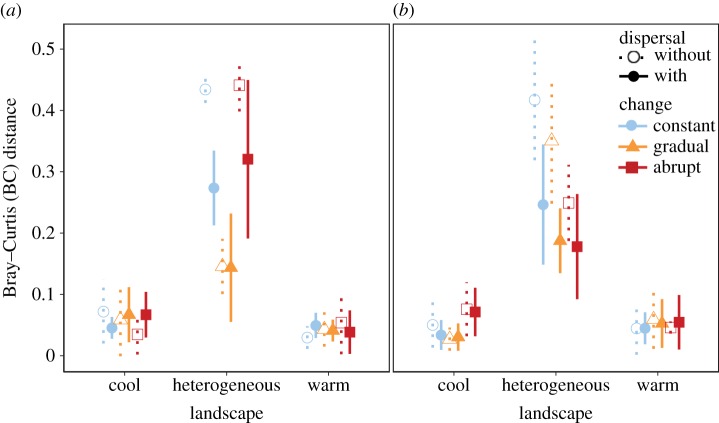
Beta diversity on (*a*) day 28 and (*b*) day 56 measured as the BC distance of the two communities of a landscape (points are the mean and bars are a standard deviation). The two habitats of a landscape were either unconnected or connected by dispersal and either remained constantly at the initial temperature or were exposed to a gradual or abrupt increase in temperature by 5°C. The landscapes were either cool, with both habitats initially at 20°C, warm, with both habitats initially at 25°C, or heterogeneous, with one cool and one warm habitat. *n* = 4 for each treatment combination. (Online version in colour.)

Diversity of our algal model communities was driven by variables related to temperature and not dispersal (figure 1). Results of the random forests are consistent with results from the GLM (electronic supplementary material, table S2). Final temperature and the rate of change were important in determining diversity. The importance of rate of change is dominated by the contrast between constant and changing environments, in particular for species richness (figure 2). When only changing environments are considered, rate of change is less important than both final and initial temperatures. Landscape heterogeneity was the most important factor in structuring beta diversity. Final beta diversity was higher in heterogeneous than in homogeneously cool or homogeneously warm landscapes (figure 3; electronic supplementary material, table S3). Dispersal did not contribute to the prediction of any of the metrics of diversity and no effect of dispersal on diversity was detected through GLM. We thus dropped dispersal as a factor in subsequent GlM analyses and focused on the effects of the rate of change, initial and final temperature on species richness and evenness.

### (b) Effects of temperature on species richness

Species richness declined with time and reached similarly low values in all treatments by the end of the experiment (figure 2*b*). However, the rate of the decline in richness differed among the treatments, resulting in significant treatment effects on day 28. The decline in species richness was mainly driven by final temperature, irrespective of the temperature history. When all habitats had reached their final temperature after 28 days, species richness and final temperature were inversely related, with the highest loss in richness occurring at the highest final temperature (figure 2*a*; GLM: final temperature: likelihood ratio test (LRT)_1,140_ = 9.99, *p* < 0.002, change: LRT_2,140_ = 0.44, *p* < 0.802). The two separate GLMs for final temperatures of 25°C and 30°C showed that temperature history had no effect on species richness (25°C: change: LRT_2,69_ = 0.058, *p* = 0.97; 30°C: change: LRT_1,46_ = 0.79, *p* = 0.374).

The effect of warming on species richness after 28 days did not depend on the initial temperature of the habitat (GLM: change × initial temperature: LRT_2,140_ = 0.44, *p* < 0.801). The decline in richness owing to warming was of similar magnitude in cool and in warm habitats, particularly when change was abrupt. Compared to habitats with constant temperature, an abrupt increase by 5°C resulted in a mean loss of 1.5 species in cool habitats and of 1.3 species in warm habitats. With a gradual change, species richness decreased on average by 1.5 and 0.7 species in cool and in warm habitats, respectively, until day 28 of the experiment.

### (c) Effects of temperature on evenness

In contrast to species richness, evenness was positively affected by final temperature after 28 days of experimental duration (figure 2*c*; ANOVA, change: *F*_2,140_ = 10.23, *p* < 0.0001; final temperature: *F*_1,140_ = 46.61, *p* < 0.0001). After 56 days, however, evenness was unaffected by final temperature (figure 2*d*; change: *F*_2,140_ = 17.65, *p* < 0.0001, final temperature: *F*_1,140_ = 0.43, *p* < 0.516). Two separate ANOVAs for final temperatures of 25°C and 30°C showed that on day 56 the type of change had a strong effect at a final temperature of 25°C (change: *F*_2,69_ = 21.26, *p* < 0.0001). Abrupt warming to 25°C resulted in higher evenness than gradual warming or constantly warm conditions. At a final temperature of 30°C, however, evenness was unaffected by the rate of change (change: *F*_1,46_ = 0.26, *p* < 0.61).

At the end of the experiment, the effect of environmental change on evenness strongly depended on the initial temperature of the habitat (figure 2*d*; change × initial temperature: *F*_2,138_ = 54.82, *p* < 0.0001). In cool habitats, warming resulted in a significant increase in evenness, but it had no effect in warm habitats. Evenness was slightly negatively correlated with total biovolume (*r*² = 0.27, *p* < 0.0001). Total biovolume was lowest after an abrupt increase in temperature from 20°C to 25°C and reached highest values in habitats of 30°C final temperature, irrespective of the rate of change (electronic supplementary material, table S6 and figure S2).

Analysis of species composition did not alter our main conclusions. Results can be found in the electronic supplementary material, tables S4, S5 and figure S3.

### (d) Response of monocultures to temperature

**Figure 4.**
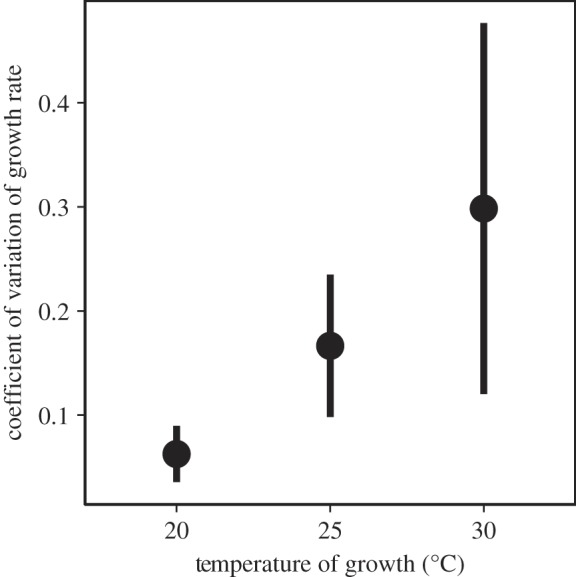
Between-species variation in growth rate at different temperatures (the error bars represent 95% confidence intervals calculated using a jackknife routine in which each species is dropped from the calculation of the coefficient of variation in turn). For each of the three final temperatures in the community experiment, variation of the growth rates between species was measured in a monoculture assay. Higher temperatures led to higher variance between species, including the inability of certain species to grow at the highest temperature.

Average growth rate was highest at the intermediate temperature of 25°C (mean_20°C_ = 0.56 d^−1^, mean_25°C_ = 0.86 d^−1^, mean_30°C_ = 0.77 d^−1^). Growth rates of five species increased with temperature, three species had their maximum growth rates at 25°C, and one species grew best at 20°C (electronic supplementary material, figure S4*a*). With increasing temperature, growth rates of species diverged so that the variance in growth rate between species increased (figure 4). Carrying capacities of most species did not change with temperature, except for those two species that were not able to grow at 30°C and except for *Scenedesmus quadricauda* which showed increasing *K* with increasing temperature (electronic supplementary material, figure S4*b*).

## 4. Discussion

Among the factors that we manipulated in our experiment, final temperature was the main driver of diversity, while habitat characteristics played only a subordinate role. Species richness declined faster at higher final temperatures, with the effect of warming depending neither on the rate of warming nor on the initial temperature of the habitats. Evenness, however, responded differently to gradual and abrupt change and the effect of warming on evenness depended on the initial temperature. At the end of the experiment, warming of warm habitats had no effect on evenness, while warming of cool habitats positively affected evenness, in particular when change was abrupt. Dispersal among cool and warm habitats did not alleviate the negative effect of rising temperature on species richness and had no effect on evenness.

### (a) Effect of final temperature

Species richness declined with time in all our treatments and reached similar values irrespective of treatment by the end of the experiment. A decline in species richness even under constant environmental conditions is a common observation in microcosm experiments with aquatic protists [31,36,37]. It was the rate of decline in richness that differed among the temperature treatments, with equilibrium richness being reached faster the higher the final temperature. A negative effect of warming on species richness or a faster decline with higher temperature has been found in a number of experiments on various groups of organisms [37–41]. Faster competitive exclusion at higher temperatures has been suggested as a possible explanation for this pattern [31]. Evidence for this hypothesis comes from experiments with cladocerans and ciliates, finding faster competitive exclusion in cladocerans [42] and higher *per capita* competitive effects with increasing temperature in ciliates [27].

Potential mechanisms behind faster competitive exclusion at higher temperatures could be either a higher average growth rate or higher variance in growth rates of species at higher temperature. Environmental conditions that result in higher population growth rates of competitors have been predicted to result in faster competitive displacement [43], such that equilibrium richness is reached faster. An alternative hypothesis is faster competitive exclusion owing to higher variance in fitness among species [44]. Our data on monoculture growth rates of the species suggest that the second of these mechanisms might be underlying the pattern of faster exclusion with higher temperatures in our model communities. While average growth rate did not steadily increase with temperature, the variation between species in their growth rates increased with higher temperatures (figure 4). Variance of growth rates, and the resulting competitive inequality, was thus a better predictor of biodiversity loss than average growth rate.

While community-level species loss in response to temperature was predictable from variation between species in their growth rates, abundances or extinction probabilities of individual species within the community could not be easily inferred from the response of species to temperature in monoculture. For most species, patch occupancy and relative biovolume along the temperature gradient did not correspond with their monoculture response (electronic supplementary material, figure S5). The pattern of a negative effect of final temperature on species richness after 28 days was driven by four species (*Gonium*, *Nitzschia*, *Pandorina* and *Pseudokirchneriella*), with all other species being either present or absent in any patch, irrespective of final temperature. These four species decreased in patch occupancy with increasing final temperature although only one of these species had consistently declining growth rate or ranking with temperature in the monoculture experiment. A characteristic that these four species had in common is that they were rare in our communities. At 20°C and 25°C each of these species contributed on average 0.4% or less to the total biovolume and declined to even lower values at 30°C, suggesting that they were poor competitors even at cool temperatures. Intensified competition at higher temperature is a possible reason for their extinction. However, competitive interactions were not strictly the only factor that differed between community cultures and monocultures, as monocultures were measured on a shorter time scale and with no gradual increase in temperature. Faster senescence at higher temperatures and thus faster extinction [45] could have influenced our results. Comparing the response of monocultures and communities to temperature on the same time scale would allow one to distinguish between effects of thermal tolerance, competitive interactions and senescence and would thus be an interesting avenue for future experiments.

Current approaches that try to predict the effect of climate change on biodiversity either use current distributions of species to project future range sizes [23] or they are based on experimentally measured thermal performance curves of species and predict the impact of climate change by comparing thermal sensitivities of species with future climate scenarios [10,11]. Neither of these two approaches takes species interactions into account. Comparison of temperature response curves of interacting species has been suggested as a way to consider species interactions in predictions on the effect of climate change [46]. Our results suggest that measurement of the divergence in growth rate between species in response to environmental change may be a useful approach to predicting biodiversity loss with environmental change.

### (b) The rate of change

The rate of environmental change did not have a long-lasting effect on the species richness of our algal model communities. Final temperature determined species richness, irrespective of the temperature history. Even in the short-term, after 28 days of the experiment, a slower rate of change did not mitigate the negative effect of warming on species richness, although communities exposed to an abrupt increase in temperature had reached the final temperature earlier than communities exposed to a gradual increase. Our results are in contrast to those of an experiment that compared the effects of a gradual versus abrupt increase in CO_2_ concentration on the diversity of a mycorrhiza fungal community [6]. In this study, an abrupt increase in CO_2_ resulted in a decline in species richness, while a gradual increase did not affect richness of mycorrhiza species compared with the ambient CO_2_ treatment. We did not find this effect for temperature although the gradual temperature increase in our experiment was applied over a similar number of generations as the gradual increase in CO_2_. Microevolutionary responses, which are supposed to be more likely under slower rates of environmental change [7,8], either did not occur in our experimental communities or were not strong enough to rescue species from extinctions. Temperature was a strong environmental filter, with final temperature overriding any effects of temperature history.

In natural communities, climate change occurs at a much slower rate than we imposed even in the gradual change treatment. However, as the algae in our experiment can have generation times shorter than a single day, the gradual increase in temperature occurred over the course of more than 24 generations. The rate of change in our experiment in terms of generations is thus comparable to that experienced by many organisms with longer generation times, such as plants, in natural settings exposed to global warming. Our results suggest that a gradual increase in temperature does not necessarily mitigate the negative effect of rising temperature.

### (c) The effect of initial temperature

By day 28 of the experiment, warming of cool habitats led to the same number of extinctions of species as did warming of already warm habitats. We had expected a stronger effect of the temperature increase in warm habitats, as warming in warm habitats would be more likely to drive species beyond their thermal optimum or tolerance limits. The monoculture growth experiment supports this hypothesis. While all species were able to grow at 20°C and 25°C, two species were unable to grow at 30°C. In addition, two species were beyond their thermal optimum at 30°C. Based on the monoculture growth results, a temperature increase in the warm habitats should thus have driven more species to extinction than a temperature increase in cool habitats. However, competitive interactions altered the thermal ranges of most species compared to the monoculture experiment. The prediction that warming has a stronger effect at warmer latitudes as species are closer to their optimum temperature is based on thermal performance curves measured in single-species experiments [10,11]. Though species at higher latitudes are farther away from their single-species thermal optimum than species at lower latitudes, they could be very close to their range limit set by species interactions. Our results add to the accumulating evidence that species interactions are key to understanding species responses to climate change [2,26]. Rising temperature can intensify competitive and trophic interactions either owing to density effects or owing to *per capita* effects [47], with density effects resulting from higher population sizes at higher temperatures and *per capita* effects resulting from higher consumption rates per individual at higher temperatures. In addition, rising temperature can disrupt species interactions owing to interspecific differences in thermal sensitivity [46,47], species-specific differences in dispersal rates resulting in different abilities to track climate change [3,46], and different rates of adaptation resulting in species-specific evolutionary responses to environmental change. Because of these altered interactions, species often do not respond to climate change as would be expected from their thermal sensitivity [46].

In contrast to natural systems, the species in our communities were not evolutionarily adapted to the initial temperatures. There is experimental evidence that the magnitude of the effect of warming on diversity may differ among communities with different evolutionary histories [22]. In addition, the two initial temperatures that we compared in our experiment differed by only 5°C, and our results might have been different when comparing habitats on a longer thermal gradient. However, the mechanism that we found to be underlying the observed pattern will probably be important also in natural systems. Communities in cool habitats could be just as sensitive to warming as communities in warm habitats, as rising temperature may result in intensified and altered species interactions.

### (d) Evenness and community composition

In contrast to species richness, the response of evenness to warming did depend on the rate of change and on the initial temperature of the habitats. By the end of the experiment, warming of cool habitats had resulted in an increase in evenness, while warming of already warm habitats had no further effect on evenness. The variation in results of studies on experimental warming, which vary from positive effects of warming on evenness to negative effects [4,31,39,48], may be best explained by the fact that the effect of warming may vary between environments with different initial conditions.

While high temperature resulted in faster extinction of inferior competitors, the loss of the rare species and the more equal contribution to the total biovolume by surviving species led to higher evenness at higher temperatures. This greater evenness between the dominant species with increasing temperature was not explained by a decrease in the variation in growth rate between these dominant species. The between-species variance in growth rate, when only the dominant species are included, also increased with temperature. The increase in final evenness owing to warming of cool habitats was especially pronounced when the increase in temperature was abrupt. Higher relative biovolume of the filamentous cyanobacterium *Anabaena* was the main reason for higher evenness in habitats that had been exposed to an abrupt increase in temperature from 20°C to 25°C than in habitats that had been gradually warmed or always been at 25°C. This high evenness after abrupt warming to 25°C corresponded with low total biovolume, as found in other experiments [38]. Among the three species that made up more than 99% of the total biovolume, *Scenedesmus acutus* was driving this pattern. Our results imply that different rates of change can have complex and long-lasting effects on community dynamics, resulting in changes in relative abundances, evenness and total biovolume.

### (e) Dispersal

The decline in species richness due to increasing temperature was not mitigated by dispersal among cool and warm habitats. Source–sink dynamics require sufficiently high beta diversity, resulting from spatial heterogeneity and regional niche partitioning of the species, and a dispersal rate that is neither too low to lead to dispersal limitation nor too high to lead to complete homogenization of the metacommunity [17]. Two mechanisms were at play that prevented source–sink dynamics from operating in our experimental metacommunities. (i) Warmer communities did not only have fewer species, but they also had no unique species and thus were only a subset of the cooler communities. Hence, no species were available in the warm habitats that would track environmental change by dispersing to the cooler habitats. (ii) Species that were not able to persist in the warmer habitats were not maintained by re-immigration from cooler habitats. Temperature acted as a strong environmental filter and prevented maintenance of inferior competitors by migration from cool to warm habitats.

While dispersal does not necessarily affect diversity at the local or regional scale in heterogeneous metacommunities, it usually tends to homogenize communities and thus decreases beta diversity [30,49]. However, we did not find a significant effect of dispersal on beta diversity. Such a lack of effect of dispersal may be owing to strong species sorting processes, which can prevent even high dispersal from homogenizing heterogeneous metacommunities [50]. A larger dispersal rate could have potentially led to a detectable effect of dispersal. We chose a dispersal rate which was similar to that used in a number of metacommunity experiments [36,38], and even very low dispersal rates can result in a decline in beta diversity [51].

Despite strong expectations of an effect of dispersal on diversity of patches and landscapes, dispersal was the least important variable in determining the diversity response to environmental change. Determination of the conditions that allow dispersal to alleviate the effect of environmental change will thus be a key to the success of conservation efforts that aim at linking habitats such as wildlife corridors. The complex interplay of dispersal and habitat heterogeneity will be of particular importance, with spatial heterogeneity determining both beta diversity and the importance of species sorting processes. In addition, the effect of dispersal will probably depend on the mode of dispersal. While we modelled a metacommunity of passively dispersed organisms, dispersal in changing environments might be particularly important for organisms that are able to actively track environmental change.

## 5. Conclusion

The results of our microcosm experiment show that temperature is a strong structuring force, with the same final temperature resulting in the same species richness, irrespective of the temperature history and irrespective of dispersal. Neither dispersal nor a slower rate of change mitigated the negative effect of warming on species richness. This highlights the need to continue the investigation of the parameter space that allows for metacommunity dynamics to mitigate the effect of environmental change, which is central to many conservation strategies. Comparison of the performance of species in full community with performance in monoculture suggests that the decline in species richness with increasing temperature was primarily owing to competitive interactions rather than thermal tolerance. Higher temperature resulted in stronger fitness inequality of species and thus faster competitive exclusion. This calls for adopting a community-level perspective when evaluating the effects of global change on species survival and distributions. Measurement of fitness inequality and divergence in growth rate with environmental change may be a promising tool in the prediction of biodiversity loss.

## Supplementary Material

ESM

## Data accessibility

Data is available on datadryad.org (doi:10.5061/dryad.6t6k6).
