# Supplementary material for: Final thermal conditions override the effects of temperature history and dispersal in experimental communities
Source: Proc Biol Sci. 2014 Oct 22;281(1793):20141540. doi: 10.1098/rspb.2014.1540 (PMC4173686; doi:10.1098/rspb.2014.1540)
Supplement: ESM [file rspb20141540supp1.pdf]

Electronic Supplementary Material to *Final thermal conditions override the effects of temperature history and dispersal in experimental communities*

Romana Limberger, Etienne Low-Décarie and Gregor F. Fussmann

1. Additional information on random forests

We estimated the relative importance of the predictor variables in explaining diversity using random forests [1]. The random forest algorithm builds multiple independent regression trees and then combines them into one model. Each tree is fit to a bootstrap sample based on two thirds of the data and each partitioning within a tree is based on a random subsample of one third of the predictor variables. The remaining samples (the out-of-bag data) are used to evaluate the accuracies of the predictions and to assess the relative importance of the predictor variables. The predictors in our model are the type of change (constant, gradual, abrupt), initial temperature (20 or 25 °C), final temperature (20, 25, 30 °C), heterogeneity (homogeneous or heterogeneous), and dispersal (with or without). We fitted 1000 regression trees to the data and evaluated the relative importance of the predictor variables in predicting diversity by using the percent increase in the mean squared error when dropping the respective predictor variable. Random forests were computed with the randomForest package in R [2].

2. Supplemental tables

Table S1: Algal species used in the experiment. Some species were obtained from the Canadian Phycological Culture Centre (CPCC) and from the Culture Collection of Algae in Göttingen (SAG), while the others were isolated from ponds in the city of Salzburg, Austria (SBG). The species represented four different taxonomic groups and varied in size and growth form. Cell volumes of the species were calculated following Hillebrand et al. [3] after measuring the dimensions of 20 cells for each species.

Species	Taxonomic group	Source	Cell volume [µm³]	Growth form
Synechococcus leopoliensis	Cyanobacteria	CPCC 102	5.14	Unicellular
Anabaena variabilis	Cyanobacteria	CPCC 105	12.28	Filament
Navicula pelliculosa	Bacillariophyceae	CPCC 552	28.20	Unicellular
Nitzschia palea	Bacillariophyceae	CPCC 160	112.10	Unicellular
Cryptomonas sp.	Cryptophyta	SAG 26.80	181.69	Unicellular
Pseudokirchneriella subcapitata	Chlorophyta	CPCC 37	37.88	Unicellular
Scenedesmus acutus	Chlorophyta	CPCC 10	56.75	Unicellular/ colonial
Scenedesmus quadricauda	Chlorophyta	SBG	77.77	Colonial
Gonium sp.	Chlorophyta	SBG	506.34	Colonial
Pandorina morum	Chlorophyta	SBG	584.17	Colonial

Table S2: Results of generalized linear models (glm) testing for treatment effects on species richness and on evenness. Species richness and evenness were calculated at the local scale (mean local richness, mean local evenness) and at the regional scale (regional richness, regional evenness). Species richness was analysed using glms with a Poisson distribution and a log link function, evenness was analysed using ANOVAs. The models tested for effects of the factors dispersal (with vs. without), change (constant, gradual, abrupt), and landscape (homogeneously cool, homogeneously warm, heterogeneous). Bonferroni correction was applied to account for the repeated sampling on two days (P < 0.025 in bold).

Mean local richness on day 28

	Df	LRT	Pr(>Chi)
Dispersal	1	0.036	0.850
Change	2	0.814	0.666
Landscape	2	0.360	0.835
Dispersal:Change	2	0.006	0.997
Dispersal:Landscape	2	0.108	0.947
Change:Landscape	4	0.228	0.994
Dispersal:Change:Landscape	4	0.048	1.000

Mean local richness on day 56

	Df	LRT	Pr(>Chi)
Dispersal	1	0.062	0.804
Change	2	0.149	0.928
Landscape	2	0.196	0.907
Dispersal:Change	2	0.003	0.999
Dispersal:Landscape	2	0.089	0.957
Change:Landscape	4	0.066	1.000
Dispersal:Change:Landscape	4	0.019	1.000

Regional richness on day 28

	Df	LRT	Pr(>Chi)
Dispersal	1	0.016	0.900
Change	2	0.493	0.782
Landscape	2	0.289	0.866
Dispersal:Change	2	0.041	0.980
Dispersal:Landscape	2	0.160	0.923
Change:Landscape	4	0.115	0.998
Dispersal:Change:Landscape	4	0.071	0.999

Regional richness on day 56

	Df	LRT	Pr(>Chi)
Dispersal	1	0.100	0.752
Change	2	0.458	0.795
Landscape	2	0.556	0.757
Dispersal:Change	2	0.014	0.993
Dispersal:Landscape	2	0.119	0.943
Change:Landscape	4	0.219	0.994
Dispersal:Change:Landscape	4	0.043	1.000

Mean local evenness on day 28

	Df	F value	Pr(>F)
Dispersal	1	0.589	0.446
Change	2	3.900	0.026
Landscape	2	1.231	0.300
Dispersal:Change	2	0.120	0.887
Dispersal:Landscape	2	0.776	0.465
**Change:Landscape**	**4**	**4.529**	**0.003**
Dispersal:Change:Landscape	4	0.287	0.885
Residuals	54		

Mean local evenness on day 56

	Df	F value	Pr(>F)
Dispersal	1	0.316	0.577
**Change**	**2**	**24.180**	**<0.0001**
**Landscape**	**2**	**17.077**	**<0.0001**
Dispersal:Change	2	0.570	0.569
Dispersal:Landscape	2	2.624	0.082
**Change:Landscape**	**4**	**13.326**	**<0.0001**
Dispersal:Change:Landscape	4	1.820	0.138
Residuals	54		

Regional evenness on day 28

	Df	F value	Pr(>F)
Dispersal	1	0.398	0.531
Change	2	2.593	0.084
Landscape	2	1.281	0.286
Dispersal:Change	2	0.045	0.956
Dispersal:Landscape	2	0.589	0.558
**Change:Landscape**	**4**	**4.425**	**0.004**
Dispersal:Change:Landscape	4	0.690	0.602
Residuals	54		

Regional evenness on day 56

	Df	F value	Pr(>F)
Dispersal	1	0.401	0.529
**Change**	**2**	**21.573**	**<0.0001**
**Landscape**	**2**	**13.512**	**<0.0001**
Dispersal:Change	2	0.164	0.850
Dispersal:Landscape	2	1.469	0.239
**Change:Landscape**	**4**	**10.574**	**<0.0001**
Dispersal:Change:Landscape	4	1.284	0.288
Residuals	54		

Table S3: Results of generalized linear models testing for treatment effects on beta diversity of the two communities of a landscape. Beta diversity was measured as Bray-Curtis dissimilarity based on the relative biovolume of species. Glms with a quasibinomial distribution and a logit link function were used to analyse the data. Bonferroni correction was applied to account for the repeated sampling on two days ($P < 0.025$ in bold).

Bray-Curtis dissimilarity on day 28

	Df	Scaled deviance	Pr(>Chi)
Change	2	2.006	0.367
**Landscape**	**2**	**92.294**	**<0.0001**
Dispersal	1	0.899	0.343
**Change:Landscape**	**4**	**15.789**	**0.003**
Change:Dispersal	2	2.417	0.299
Landscape:Dispersal	2	3.426	0.180
Change:Landscape:Dispersal	4	4.241	0.374

Bray-Curtis dissimilarity on day 56

	Df	Scaled deviance	Pr(>Chi)
Change	2	3.457	0.178
**Landscape**	**2**	**84.509**	**<0.0001**
Dispersal	1	0.464	0.496
Change:Landscape	4	8.406	0.078
Change:Dispersal	2	0.357	0.837
Landscape:Dispersal	2	1.617	0.445
Change:Landscape:Dispersal	4	0.504	0.973

Table S4: Results of MANOVAs testing for treatment effects on species composition. Relative biovolume of species was square-root transformed prior to analyses for normality. The model tested for effects of the factors dispersal (with vs. without), change (constant, gradual, abrupt), and landscape (homogeneously cool, homogeneously warm, heterogeneous). Bonferroni correction was applied to account for the repeated sampling on two days ($P < 0.025$ in bold).

Species composition on day 28

	Df	approx F	Pr(>F)
**Change**	**2**	**23.512**	**<0.0001**
**Landscape**	**2**	**34.693**	**<0.0001**
Dispersal	1	0.784	0.604
**Change:Landscape**	**4**	**6.643**	**<0.0001**
Change:Dispersal	2	0.377	0.979
Landscape:Dispersal	2	0.728	0.742
Change:Landscape:Dispersal	4	0.961	0.526
Residuals	54		

Species composition on day 56

	Df	approx F	Pr(>F)
**Change**	**2**	**12.061**	**<0.0001**
**Landscape**	**2**	**19.316**	**<0.0001**
Dispersal	1	0.582	0.767
**Change:Landscape**	**4**	**3.450**	**<0.0001**
Change:Dispersal	2	0.634	0.831
Landscape:Dispersal	2	1.168	0.312
Change:Landscape:Dispersal	4	0.871	0.656
Residuals	54		

Table S5: Results of ANOVAs testing for main effects of the factors change and final temperature on the relative biovolume of species at the end of the experiment. We dropped the factor dispersal as the MANOVA had found no effect of dispersal on species composition (Table S3). Results are shown for the three dominant species that together made up more than 99% of the total biovolume. Relative biovolume of species was square-root transformed prior to analyses.

Anabaena

	Df	F value	Pr(>F)
**Change**	**2**	**26.611**	**<0.0001**
**Final temperature**	**1**	**134.268**	**<0.0001**

Synechococcus

	Df	F value	Pr(>F)
**Change**	**2**	**11.863**	**<0.0001**
**Final temperature**	**1**	**625.456**	**<0.0001**

Scenedesmus acutus

	Df	F value	Pr(>F)
**Change**	**2**	**23.707**	**<0.0001**
**Final temperature**	**1**	**804.124**	**<0.0001**

Table S6: Results of an ANOVA testing for effects of the factors change and initial temperature on the total biovolume at the end of the experiment. We dropped the factor dispersal which had no effect when included in the model. Total biovolume was log-transformed prior to analyses. Warming of cool habitats resulted in a decline in total biovolume, but only when warming was abrupt. Warming of warm habitats resulted in an increase in total biovolume irrespective of the rate of warming.

Total biovolume on day 56

	Df	F value	Pr(>F)
**Change**	**2**	**16.245**	**<0.0001**
**Initial temperature**	**1**	**10.332**	**0.002**
**Change:Initial temperature**	**2**	**34.86**	**<0.0001**

3. Supplemental Figures

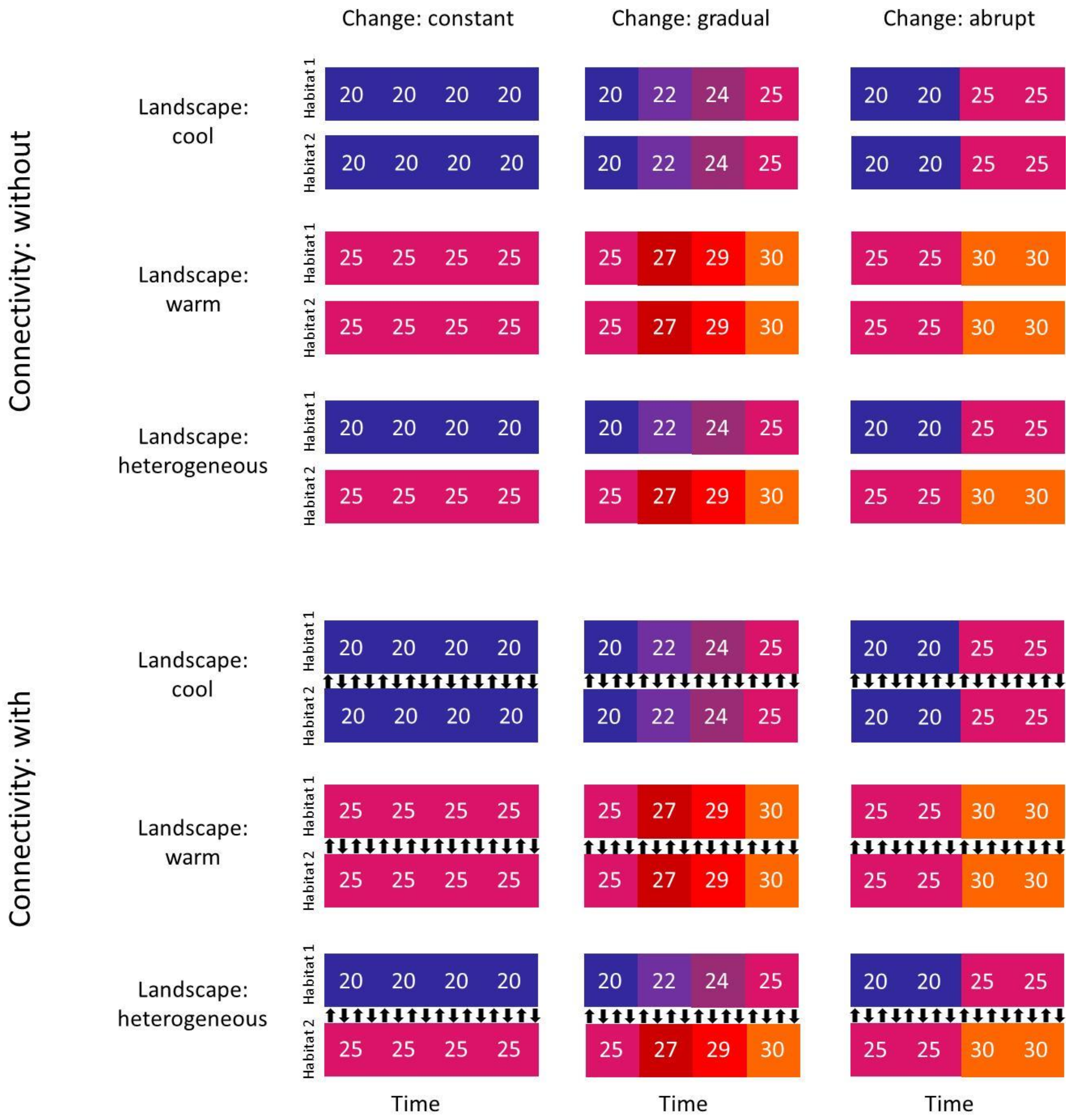

Fig. S1: Scheme of the experimental design. Each box represents a habitat (i.e. a microcosm) and shows how temperature (in °C) changed with time in the respective habitat. Temperature either remained constantly at the initial temperature or was gradually or abruptly increased by 5 °C. The gradual increase started on day 4 of the experiment and ended on day 28, while the abrupt increase was imposed on day 16, resulting in the same mean temperature for gradual and abrupt increase. All habitats were then maintained for another 28 days at the final temperature. Initial temperature in the habitats was either 20 °C or 25 °C. Two habitats each formed a landscape, with either both habitats initially at 20 °C (cool landscapes), or both habitats initially at 25 °C (warm landscapes), or one cool and one warm habitat forming a landscape (heterogeneous landscapes). The two habitats of a landscape were either unconnected or connected by dispersal. Each treatment was replicated 4 times.

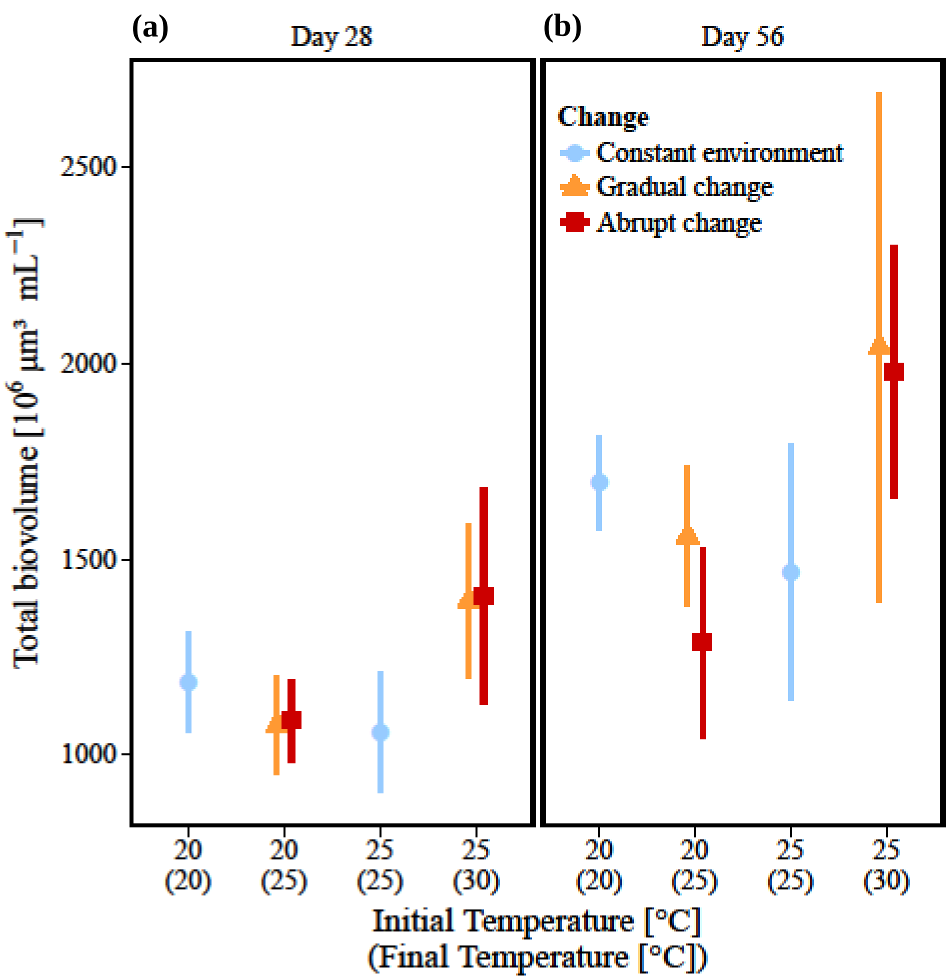

Fig. S2: The effects of the rate of change, initial and final temperature on total biovolume on days 28 and 56 of the experiment. Communities were either kept at a constant temperature or were exposed to a gradual or abrupt increase by 5 °C. Initial temperature of the habitats was 20 or 25 °C, resulting in final temperatures of 20, 25, and 30 °C, respectively. Data from connected and unconnected habitats were pooled; points are the mean and bars are a standard deviation. N = 24 for each treatment combination.

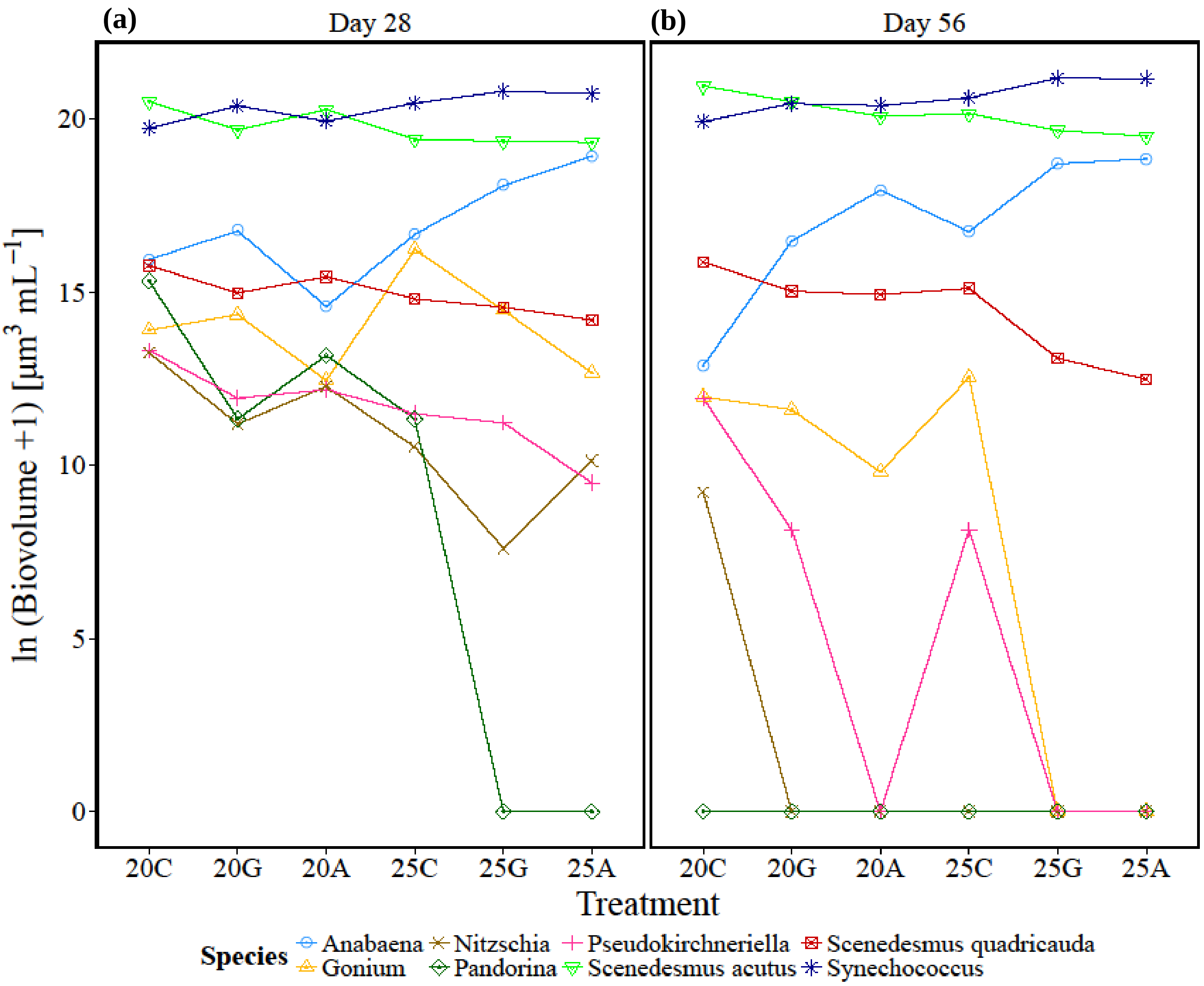

Fig. S3: Species composition in habitats with constant temperature (C), with gradually increasing temperature (G), and with abruptly increasing temperature (A). Initial temperature was either 20 or 25 °C. By day 28, two species had died out in any patch; only the remaining eight species are shown. N = 24 for each treatment combination. *Synechococcus*, *Anabaena*, and *Scenedesmus acutus* were the dominant species, together making up more than 99 % of the total biovolume in any patch at the end of the experiment. Increasing final temperature resulted in a decrease in the biovolume of the chlorophyte *Scenedesmus acutus* and in an increase in the biovolume of the cyanobacteria *Synechococcus* and *Anabaena*. Most of the rare species declined in biovolume with increasing final temperature.

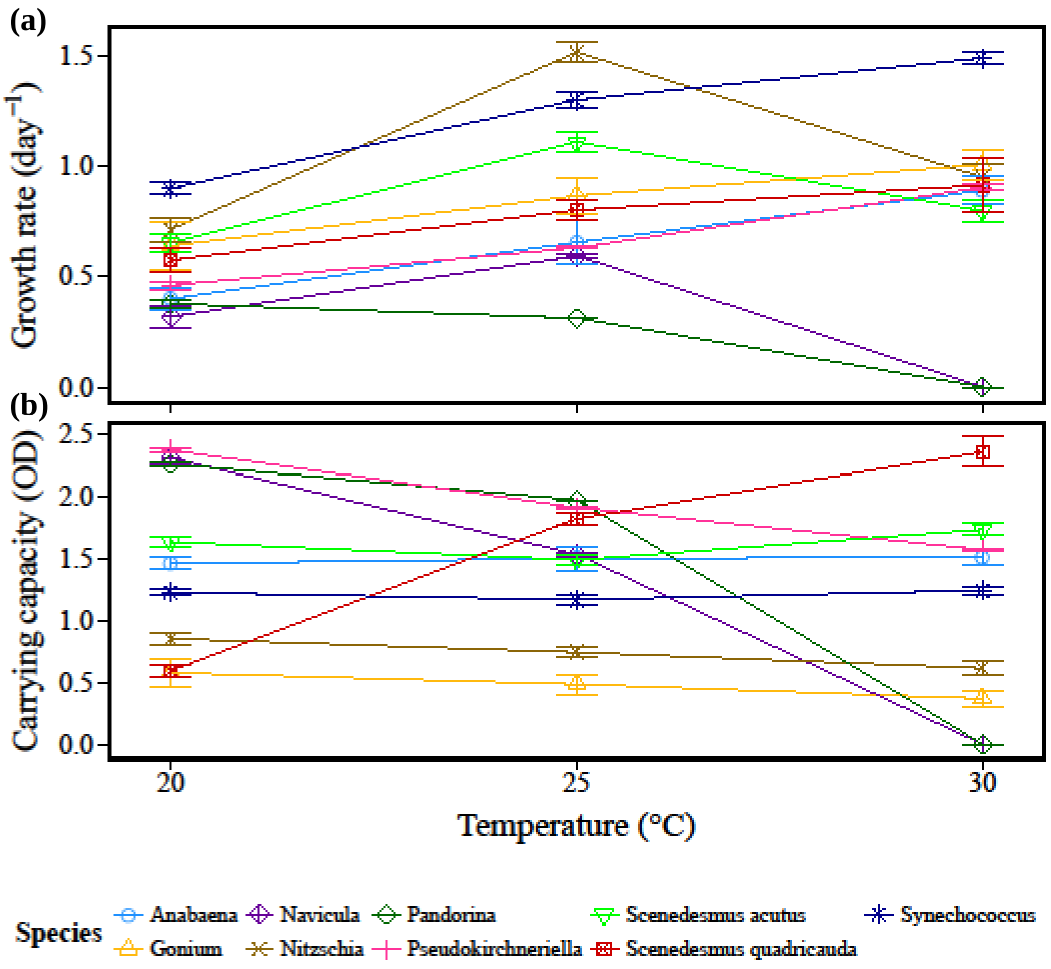

Fig. S4: Growth rates [day^{-1}] and carrying capacities [optical density] of species at 20, 25, and 30 °C measured in a single-species experiment (mean ± SE, N = 3). Carrying capacities are inferred from growth curves that have at least passed the inflection point, but may not have reached full carrying capacity for slow growing species. The comparatively high carrying capacities of *Pseudokirchneriella*, *Pandorina* and *Navicula* at 20 °C are likely an overestimation as growth curves had not reached full carrying capacity.

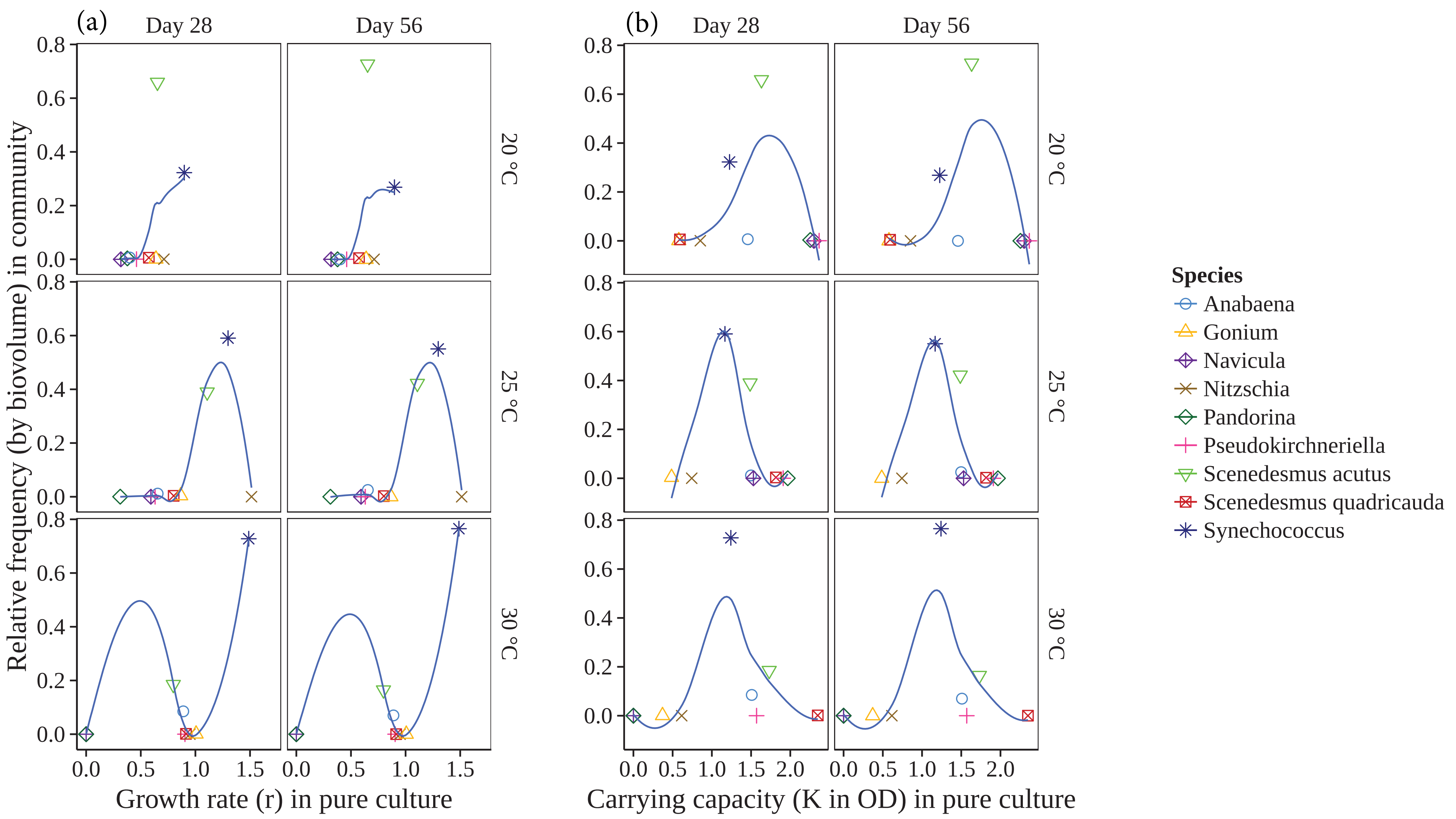

Fig. S5: Relative frequency of species in the community experiment versus (a) growth rate [day-1] and (b) carrying capacity [optical density] of the species in monoculture. Growth rates and carrying capacities were measured at 20, 25, and 30 °C, the three final temperatures of the community experiment. Relative frequency in the communities was averaged over cultures experiencing the same final temperature. The curves are locally weighted scatterplot smoothing fits to the data. Species that failed to grow at 30 °C also failed to grow in the community at this temperature, while high r or K at a given temperature did not necessarily result in dominance in the community at the respective temperature.

References

1. Breiman L. 2001 Random forests. *Machine Learning* **45**, 5-32. (doi:10.1023/a:1010933404324).
2. R Development Core Team. 2009 R: A language and environment for statistical computing. R Foundation for Statistical Computing. Vienna, Austria.
3. Hillebrand H., Durselen C.D., Kirschtel D., Pollingher U., Zohary T. 1999 Biovolume calculation for pelagic and benthic microalgae. *J Phycol* **35**, 403-424. (doi:10.1046/j.1529-8817.1999.3520403.x).
